# Oxidative stress facilitates exogenous mitochondria internalization and survival in retinal ganglion precursor-like cells

**DOI:** 10.1038/s41598-022-08747-3

**Published:** 2022-03-24

**Authors:** Michal Aharoni-Simon, Keren Ben-Yaakov, Maya Sharvit-Bader, Daniel Raz, Yasmin Haim, Waleed Ghannam, Noga Porat, Hana Leiba, Arie Marcovich, Avital Eisenberg-Lerner, Ziv Rotfogel

**Affiliations:** 1 Ophthalmology Research Laboratory, Kaplan Medical Center, 76100 Rehovot, Israel; 2 Faculty of Medicine, Hadassah Medical School, The Hebrew University of Jerusalem, Jerusalem, Israel

**Keywords:** Cell biology, Medical research

## Abstract

Ocular cells are highly dependent on mitochondrial function due to their high demand of energy supply and their constant exposure to oxidative stress. Indeed, mitochondrial dysfunction is highly implicated in various acute, chronic, and genetic disorders of the visual system. It has recently been shown that mitochondrial transplantation (MitoPlant) temporarily protects retinal ganglion cells (RGCs) from cell death during ocular ischemia. Here, we characterized MitoPlant dynamics in retinal ganglion precursor-like cells, in steady state and under oxidative stress. We developed a new method for detection of transplanted mitochondria using qPCR, based on a difference in the mtDNA sequence of C57BL/6 and BALB/c mouse strains. Using this approach, we show internalization of exogenous mitochondria already three hours after transplantation, and a decline in mitochondrial content after twenty four hours. Interestingly, exposure of target cells to moderate oxidative stress prior to MitoPlant dramatically enhanced mitochondrial uptake and extended the survival of mitochondria in recipient cells by more than three fold. Understanding the factors that regulate the exogenous mitochondrial uptake and their survival may promote the application of MitoPlant for treatment of chronic and genetic mitochondrial diseases.

## Introduction

Mitochondrial dysfunction contributes to a wide range of pathologies. In addition to intracellular morphological and positional changes of the mitochondria in response to stress, it has been reported that mitochondria can be transferred between cells and protect damaged cells from death^1^. Transfer of mitochondria from astrocytes to cerebral neurons after ischemia has been shown to improve neuron viability and the prognosis of the animals^2^. Moreover, mitochondrial transfer between cancerous cells has been shown to increase their tumorigenic potential and chemoresistance^3,4^.

Islam et al. demonstrated the therapeutic potential of mitochondrial transfer by showing that instillation of mouse bone marrow-derived stromal cells (mBMSC) into acutely injured mouse lungs resulted in transfer (‘donation’) of mitochondria into the injured tissue and increased cell survival after injury^5^. A similar strategy for protecting damaged tissue is to provide freshly isolated, healthy, mitochondria into damaged tissue by a process named mitochondrial transplantation (MitoPlant).

As early as 1982, successful MitoPlant was demonstrated in vitro when Clark and Shay found that simple co-incubation of isolated mitochondria with mammalian cells resulted in spontaneous internalization of the mitochondria by the cells^6^. Since then, there are accumulated evidence that MitoPlant has a protective effect, mainly in acute, ischemic insults. Several groups have shown beneficial effects of MitoPlant in spinal cord injury^7^; hepatic ischemia^8^; axonal degeneration^9^; as well as in rodent models of schizophrenia and Parkinson’s disease^10,11^. Recently, McCully's group utilized this technique for the recovery of myocardial dysfunction in a small group of pediatric patients suffering from congenital heart disease^12^. Previous studies have shown that the transplanted mitochondria are functional inside recipient cells; increasing oxygen consumption, ATP production, calcium buffering capacity, cell proliferation, and survival, and can replace dysfunctional mitochondria in Rho zero (ρ^0^) cells, which lack mtDNA^13–15^. However, the mechanism of MitoPlant is not clear and many questions remain to be answered with regards to the factors that affect the efficiency and dynamics of mitochondrial uptake, as well as the activity and survival of transplanted mitochondrial following internalization into the recipient cells.

The visual system is one of the most energy requiring biological systems. As such, it is highly susceptible to impaired energy supply caused by mitochondrial dysfunction. Various chronic and acute ischemic insults, such as central retinal artery occlusion^16^, ischemic optic neuropathy^17,18^ and acute angle closure glaucoma^19^, typically induce deterioration in mitochondrial function. Consequently, this leads to cell death of retinal ganglion cells (RGCs), which are responsible for the transmission of the visual information from the retina to the brain. Jiang et al.^20^ demonstrated that injection of induced pluripotent stem cell derived-MSCs into the vitreal cavity of Ndufs4-KO mice resulted in mitochondrial donation to RGCs and increased their survival.

Moreover, Nascimento-dos-santos et al.^21^ demonstrated that active isolated mitochondria that are intravitraelly injected into a mouse eye are internalized by RGCs and protect them from ischemic cell death. However, the protective effect was observed only after 14 days, while after 28 days the number of RGCs was identical in all study groups, suggesting that the protective effect was temporary.

Oxidative stress is another critical insult in the visual system and is a trigger of prevalent retinal pathologies like AMD, glaucoma, and diabetic retinopathy^22^. While most ocular tissues are exposed to oxidative stress, cells with a high metabolic rate, like photoreceptors and retinal ganglion neurons, are particularly sensitive to oxidative damage^22^. We therefore sought to examine mitochondrial transplantation in retinal ganglion precursor-like cells, focusing on the dynamics and the potential effect of oxidative stress on mitochondrial uptake and survival. By developing a new research tool that enables following exogenous mitochondria within recipient cells, we demonstrate that oxidative stress significantly enhances exogenous mitochondria uptake and survival in the retinal ganglion precursor-like cell line 661W. These results promote the understanding of MitoPlant mechanisms and may contribute to the development of tools to prolong mitochondrial survival, thereby offering novel opportunities for the use of MitoPlant as a promising therapy for chronic and genetic mitochondrial diseases.

## Methods

### Animals

C57BL/6 and BALB/c inbred mice aged 8–12 weeks, purchased from Envigo, Israel, were used for this study. The mice were housed in the Kaplan Medical Center animal facility at 21–22 °C with 12/12-h light–dark cycles. Prior to dissection of livers for mitochondrial isolation, mice were fasted over-night, anesthetized using CO_2_ and euthanized. All animals received humane care, and all study protocols were approved by the Israeli National Council for Animal Experimentation (permit number IL-18-8-2018). The study was performed in compliance with the ARRIVE guidelines (https://arriveguidelines.org/). All methods were performed in accordance with the relevant guidelines and regulations.

### Cell culture

661W cells were a generous gift from Prof. Muayyad Al-Ubaidi (University of Houston, TX, USA). The 661W cell line is derived from a C57BL/6 mouse retinal tumor, and is characterized by expression of retinal photoreceptors/ganglion precursor-like markers^23,24^. 661W cells were grown in Dulbecco's Modified Eagle's Medium (DMEM) supplemented with 10% fetal bovine serum (FBS), 1 g/L glucose, 2 mM l-glutamine, 200 U/mL penicillin and 200 mg/mL streptomycin in a humidified atmosphere of 5% CO_2_ at 37 °C. For hydrogen peroxide (H_2_O_2_) experiments, cells were seeded in cell culture dishes and allowed to adhere overnight. Then, cells were exposed to H_2_O_2_ (0.1–1 mM, as indicated in the figure legends) for 1 hour, followed by removal of H_2_O_2_ and washing with PBS before addition of complete media, either with or without isolated mitochondria. Propidium Iodide exclusion was measured by flow cytometry following H_2_O_2_ treatment for evaluation of membrane integrity.

### Mitochondrial isolation

Mitochondria were isolated from C57BL/6 or BALB/c mouse livers according to published protocols^25,26^. In brief, the livers were washed with ice-cold IB-1 buffer (225 mM mannitol, 75 mM sucrose, 30 mM Tris–HCl, 0.5% fatty acid free bovine serum albumin (FA-free BSA), 0.5 mM EGTA, pH 7.4) and homogenized to break the cellular membrane. The homogenate was centrifuged twice at 750 g for 10 min at 4 °C, and the supernatant was separated and centrifuged at 10,000*g* for additional 10 min at 4 °C. The supernatant containing the cytosolic fraction was kept for western blot (WB) analysis. The pellet, which contained the mitochondria, was gently re-suspended in IB-2 buffer (same as IB-1 but without EGTA) and filtered through 10 µM membrane. The mitochondria were kept on ice and used within 3–4 hours.

### ATP production

Isolated mitochondria were diluted 1:100 in respiration buffer comprising 225 mM mannitol, 75 mM sucrose, 2 mM HEPES, 10 mM KH2PO4, 5 mM MgCl2, 1 mM EGTA and 0.1% FA-free BSA, pH 7.4. Succinate (5 mM) was added as an electron-donor to all vials. Basal ATP production, ADP-induced respiration and ATP production (150 µM ADP as substrate) were determined using ATPlite™ luminescence assay kit (PerkinElmer^®^, US) according to manufacturer instructions. Oligomycin (10 µM), an inhibitor of ATP synthase, was used as a control to validate mitochondrial-dependent ATP production. Luminescence was measured by a microplate reader (Tecan, Switzerland).

### Mitochondrial respiration

Oxygen consumption studies were performed using a Clark type electrode (Hansatech Instruments, UK) as described in Rustin et al.^27^, and according to the manufacturer instructions. Isolated mitochondria (1–2 mg) were measured in a respiration buffer at 37 °C. Succinate (10 mM), Malate (1 mM), and Sodium Pyruvate (5 mM) were used as substrates, which feed electrons into the respiratory chain via complexes I and II (state II respiration). To induce state III (complex III) respiration, 3 mM ADP was added to the chamber, and oxygen consumption was recorded for a few minutes until addition of 3.3 µM oligomycin to induce state IV respiration. Finally, 10 µM FCCP were added to confirm that the mitochondria are coupled and functional.

### Flow cytometry for evaluating cellular mitochondrial content

To evaluate mitochondrial internalization into 661W cells, 70,000 cells were seeded in a twelve-well plate. The day after, mitochondria were isolated from C57BL/6 livers, stained with MitoTracer Green FM (MTG) and the mitochondria were added to the wells in a 1:10–1:50 ratio. At different time points, the cells were detached from the wells with trypsin, centrifuged and re-suspended in PBS. Mitochondrial internalization was evaluated (excitation at 488 nm and emission at 560 nm) and the mean GFP fluorescence intensity in the cells was determined using CytoFLEX (Beckman Coulter, IN, USA). Dead cells were excluded from analysis using DRAQ7 probe (excitation at 488 nm and emission at 690 nm). For MitoPlant dynamics experiments, the laser settings were fixed throughout the days. As positive control, on the day of MitoPlant, endogenous mitochondria were stained with MTG. To evaluate the decline in GFP fluorescence due to cell proliferation, MTG fluorescence intensity was determined daily for the endogenous stained mitochondria.

### Microscopy for assessing mitochondrial uptake

661W cells in complete medium were seeded on cover-slips in 24 well plates (50,000 cells/well) a day before mitochondrial transplantation. Isolated mitochondria were stained with 400 nM MitoTracker-Red CMXRos (MTR) for 30 min at 37 °C, followed by X3 washes with PBS. Stained mitochondria were incubated for 24 hours with 661W cells. Then, the cells were fixed with 4% PFA for 10 min, and stained with TOMM20 antibody (Abcam # ab186735, 1:500) to detect endogenous mitochondria, and DAPI. Exogenous mitochondria were evaluated by MTR fluorescence. Pictures were acquired using Nikon eclipse Ts2 microscope (Nikon Instruments Inc., NY, USA) or Leica SP8 LIGHTING confocal microscope (Wetzlar, Germany).

### Assessing mitochondrial survival using qPCR

The transplanted mitochondria survival in the acceptor cells was evaluated by qPCR. For this, specific primers for polymerase chain reaction (PCR) relying on the differences between the mtDNA sequences of C57BL/6 and BALB/c mice were designed. Primers that recognize C57BL/6 mtDNA specific sequence do not recognize BALB/c mtDNA sequence, and vice versa. Half a million 661W cells were seeded in 10 cm plates and the day after the cells were transplanted with mitochondria. Cells were collected every 24 hours. For the 72–96 hours time points, cells were split to avoid over growth and death of the cells. Cellular DNA was extracted using DNeasy Blood &Tissue kit (Qiagen) according to the manufacturer protocol. For qPCR, 300 ng DNA were loaded into qPCR plate, and mixed with primers for BALB/c mtDNA and SYBR Green and the DNA was amplified using QuantStudio1 instrument (Applied Biosystems, Thermo Fisher Scientific (MA, USA). The results were normalized to D-loop and mouse mtDNA sequence that is identical in both mice mtDNA sequence. In the MitoPlant dynamics studies, the results were also normalized to the positive control sample of each day (50 million isolated mitochondria that were mixed with control cells of the same experimental time point). Primer sequences: BALB/c F′: CTGACATTTTGTAGACGTAA, R′: GAAGATAACAGTGTACAGGTTG. C57BL/6 F′: CTGACATTTTGTAGACGTAG R′: GAAGATAACAGTGTACAGGTTA. D-loop: F′: GGTTCTTACTTCAGGGCCATCA; R′: GATTAGACCCGATACATCGAGAT; mtDNA: F′: CCCAGCTACTAC ATCATTCAAGT R′: GATGGTTTGGGAGATTGGTTGATGT.

### Statistical analyses

Data is presented as mean ± SE. Data was collected from at least three independent experiments for statistical analysis. Differences between two groups were evaluated by unpaired Student’s t test, and multiple groups by one-way ANOVA followed by Bonferroni multiple comparisons test. In the flow cytometry, ATP, and qPCR studies, where the data is normalized to control sample, the data was z-transformed prior to analysis in order to enable statistical comparison. Statistical analysis was performed using GraphPad version 8.0.2 (San Diego, CA). All Differences are considered significant if p< 0.05.

## Results

### Mitochondrial isolation and characterization

**Figure 1:**
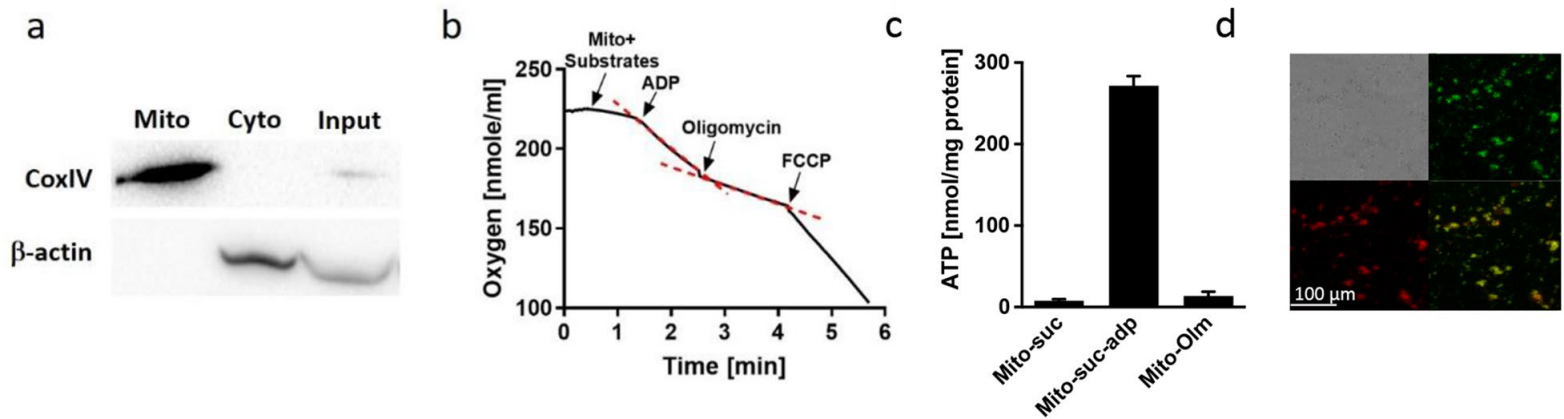
Mitochondrial isolation and characterization. Mitochondria were isolated from mouse liver. (**a**) Western blot analysis showing a clean mitochondrial fraction (mito), enriched with the mitochondrial marker CoxIV and depleted of the cytosolic marker β-actin, which appears in the cytosolic fraction (cyto). Both markers appear in the input sample (before fractionation). (**b**) Oxygen level measurement showing increased consumption after addition of mitochondrial substrates (10 mM Succinate, 1 mM Malate and 5 mM Sodium Pyruvate) and 3 mM ADP, decreased rate after addition of 3.3 µM Oligomycin and maximal consumption after addition of 10 µM FCCP. Red dotted lines exemplify the slope of the reaction. (**c**) ATP levels are increased following addition of ADP substrate (Mito-ADP) and decreased when ATP-synthase inhibitor oligomycin is added (Mito-Olm). Mitochondria alone showed base-line ATP production (Mito-suc). (**d**) Isolated mitochondria were stained with MTR, a marker for active mitochondria (with appropriate membrane potential, red) and with MTG, a marker for pan mitochondria (green). Almost all mitochondria were stained with both dyes, indicating existent membrane potential.

We have isolated mitochondria for transplantation from mouse liver and evaluated their purity and quality (Fig. 1, Supplementary Fig. 1). As shown in Fig. 1a, the purified mitochondrial fraction was enriched with the mitochondrial protein Cytochrome c oxidase (CoxIV) and was depleted of the cytosolic protein β-actin, demonstrating a highly enriched mitochondrial preparation. Next, we examined mitochondrial respiration by measuring oxygen consumption using a Clark oxygen electrode. Oxygen consumption rate was increased after addition of ADP (state 3 respiration) and decreased following addition of the ATP Synthase inhibitor Oligomycin (state 4), producing a respiratory control ratio (RCR) of 3.1. The uncoupler FCCP induced oxygen consumption, altogether indicating that the mitochondria were coupled and functional (Fig. 1b). We then confirmed mitochondrial activity by measuring ATP levels. As expected, addition of ADP increased ATP production, while introduction of oligomycin reduced ATP levels (Fig. 1c). Finally, isolated mitochondria were labeled with MitoTracker Green FM (MTG), which stains mitochondria regardless of their membrane potential, as well as with MitoTracker Red CMXRos (MTR), which only accumulates in mitochondria with intact membrane potential. As shown in Fig. 1d, almost all mitochondria were stained with both dyes, indicating appropriate membrane potential. These results demonstrate the successful isolation of clean and active mitochondria.

### In vitro uptake of isolated mitochondria by retinal ganglion precursor-like cells

**Figure 2:**
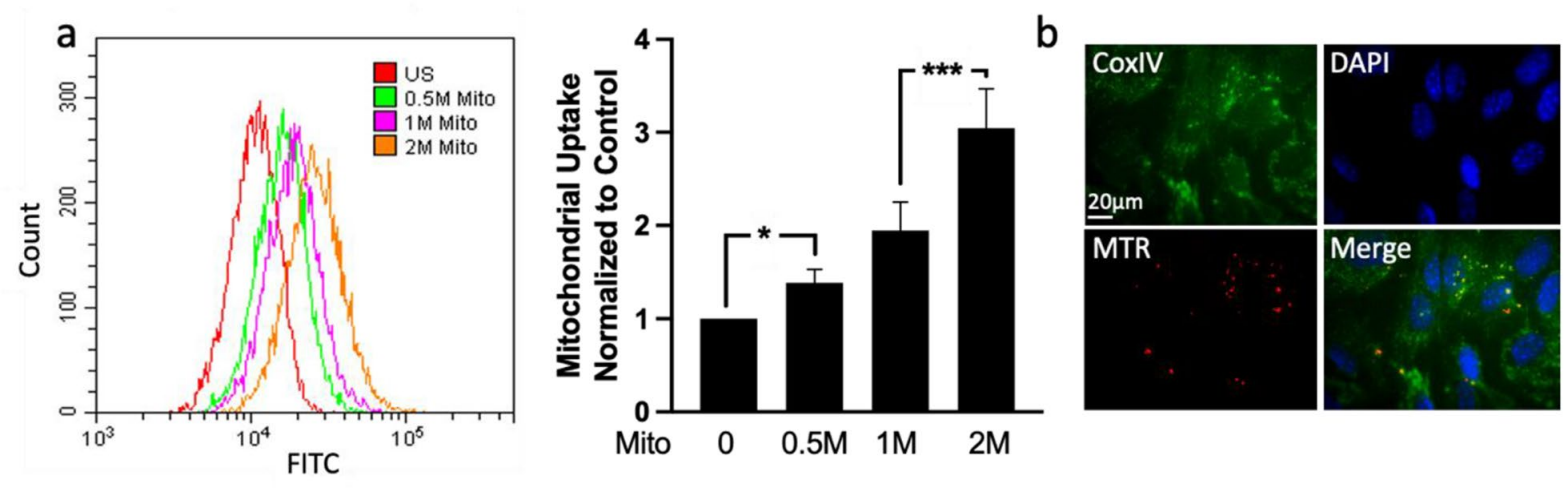
In vitro uptake of isolated mitochondria by retinal ganglion precursor-like cells. Isolated mitochondria from mouse liver were stained with mitotracker green (MTG) (**a**) or mitotracker red (MTR) (**b**) and transplanted into 100,000 661W cells during 18–24 hours of incubation. (**a**) Flow cytometry analysis of 661W cells transplanted with exogenous mitochondria showed an increase in fluorescence compared to control unstained cells, as demonstrated by a representative histogram plot (left) and by the quantitative bar graph (right), n = 4. *p < 0.05; ***p < 0.0003; *Mito* mitochondria, *M* million. (**b**) Immunofluorescence staining shows mitochondria (red) uptake by 661W cells, stained for CoxIV (endogenous mitochondria marker, green) and for the nuclear marker DAPI (blue).

Having demonstrated the isolation of active mitochondria, we tested whether isolated mitochondria can enter C57BL/6 mice-derived 661W retinal ganglion precursor-like cells. Isolated mitochondria were labeled with MTG (for flow cytometry analysis) or with MTR (for imaging analysis), and incubated with 661W cells for 18–24 hours. Flow cytometry analysis demonstrated a dose-dependent increase in exogenous mitochondria content in recipient 661W cells (Fig. 2a). The uptake of mitochondria was also observed using fluorescence microscopy which confirmed the internalization of exogenous mitochondria by the cells, in close proximity to endogenous mitochondria (Fig. 2b).

### A new assay to assess exogenous mitochondrial survival using qPCR

**Figure 3:**
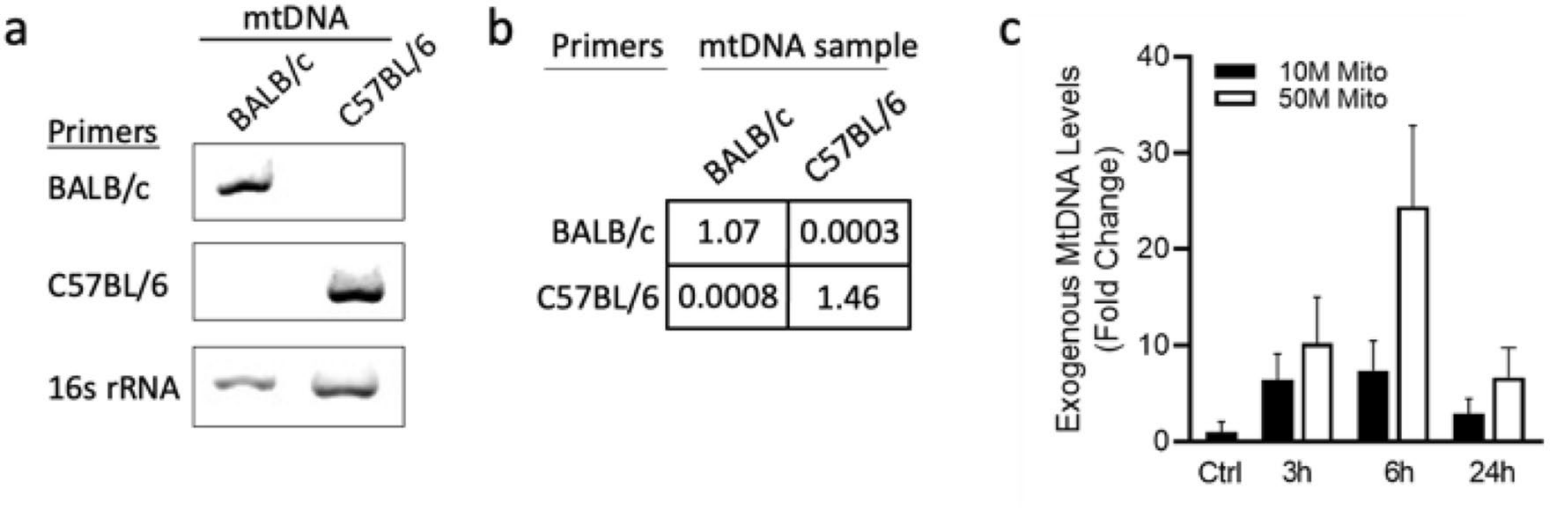
Assessment of exogenous mitochondrial survival by qPCR. (**a**) mtDNA from either BALB/c or C57BL/6 mice was amplified by PCR using primers specific for BALB/c or C57BL/6, or against 16S, a common target, as indicated. (**b**) qPCR analysis using specific primers for mtDNA from BALB/c and C57BL/6. Levels were normalized to D-loop (an identical sequence in both mtDNA sequences) and are represented as the relative quantification of the specific primers. (**c**) Survival rate of the transplanted mitochondria. One million 661W cells (C57BL/6 origin) were transplanted with either 10 million or 50 million (M) mitochondria that were isolated from BALB/c mouse liver. After 3, 6, or 24 hours, RNA was extracted from the cells and presence of exogenous BALB/c mitochondria was assessed by qPCR. n = 4.

In order to evaluate exogenous mitochondria survival in cells following transplantation, we designed an approach for differential detection of mitochondria from C57BL/6 and BALB/c mice using polymerase chain reaction (PCR). Specifically, we designed primers that distinct between mitochondria from these strains based on a difference in two nucleotides in their mitochondrial DNA (mtDNA) sequence^28^. As shown in the PCR results (Fig. 3a, Supplementary Fig. 2), BALB/c-specific primers amplified only mtDNA isolated from BALB/c liver mitochondria and not C57BL/6 mtDNA, and vice versa. Both mtDNA sequences were amplified by primers targeting common sequences of the two strains (16S and mtDNA). The specificity of the primers for distinction of mitochondria from C57BL/6 versus BALB/c was further confirmed using qPCR (Fig. 3b). Then, we applied this approach to evaluate the survival of exogenous mitochondria (from BALB/c mouse liver) in 661W cells (which are originated from C57BL/6 mice) using qPCR. As shown in Fig. 3c, mitochondrial uptake and survival is time- and dose-dependent. More exogenous mitochondria can be detected in 661W cells transplanted with 50 million compared to 10 million mitochondria, indicating the sensitivity of the assay to detect differences in the input number of transplanted mitochondria. In addition, exogenous mitochondria could be detected in the cells already after 3 hours, peaked after 6 hours and then declined at 24 hours, suggesting that the transplanted mitochondria survive in host cells under steady state conditions for approximately 24 hours.

### Oxidative stress facilitates mitochondrial uptake by retinal ganglion precursor-like cells

**Figure 4:**
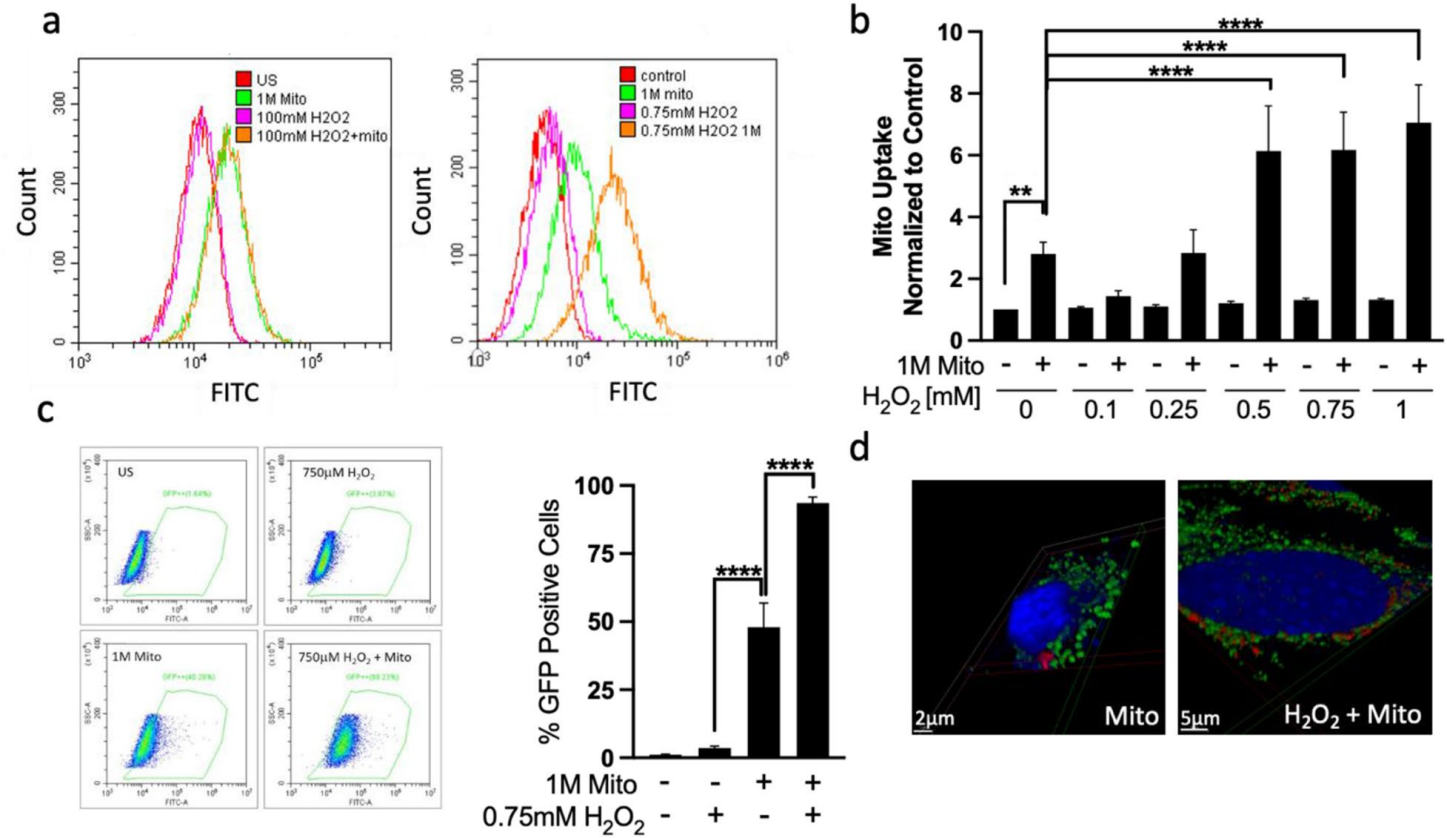
Oxidative stress facilitates mitochondrial uptake by retinal ganglion precursor-like cells. Cells were exposed to increasing concentrations (as indicated) of H_2_O_2_ for 1 hour and then transplanted with 1 million (M) MTG-stained mitochondria. Mitochondrial internalization was determined by flow cytometry 24 hours following transplantation. Enhanced mitochondrial uptake by H_2_O_2_ concentrations higher than 0.5 mM is demonstrated by representative histogram plots (**a**) and by a quantitative bar graph (**b**). n = 4, **p < 0.005 compared to control, non-transplanted cells; ****p < 0.0001 compared to mitochondria treatment group. (**c**) The percentage of cells that contain exogenous mitochondria (GFP Positive population) is demonstrated by representative histogram plots (left) and by a quantitative bar graph (right). n = 6, * p < 0.0001. (**d**) Three dimensional view of confocal planes, showing close localization of exogenous mitochondria (red, MTR-stained mitochondria) and endogenous mitochondria (green, stained with TOMM20 Ab).

Since previous studies showed enhanced mitochondrial transfer following ischemia^2–4^, we next sought to examine whether moderate oxidative stress facilitates mitochondrial uptake in retinal ganglion precursor-like cells. To this end, 661W cells were exposed to increasing concentrations of hydrogen peroxide (H_2_O_2_) for 1 hour, followed by transplantation of mitochondria that were pre-stained with mitotracker dyes. Twenty-four hours following transplantation, mitochondrial internalization was analyzed by flow cytometry and confocal microscopy (Fig. 4). While 0.1–0.25 mM H_2_O_2_ did not affect mitochondrial uptake, 0.5–1 mM H_2_O_2_ significantly enhanced mitochondrial internalization, as demonstrated by quantitative analysis of flow cytometry results (Fig. 4a,b), and by confocal microscopy imaging (Fig. 4c). We further confirmed that these experimental conditions did not induce cell death by evaluating membrane permeabilization using propidium iodide (PI) staining in flow cytometry (Supplementary Fig. 3). We chose to work with 0.75 mM H_2_O_2_ for the rest of the experiments as this concentration efficiently enhanced mitochondria uptake without compromising membrane integrity. Finally, we monitored that transferred mitochondria remain intact and functional in recipient 661W cells. To this end, we examined mitochondrial membrane potential, a hallmark of functional mitochondria, reflecting the activity of the mitochondrial proton pumps (Complexes I, III and IV) that are required to generate ATP. We stained isolated mitochondria with MTR, a dye which only accumulates in mitochondria with intact membrane potential, then transferred the stained mitochondria into recipient 661W cells, and examined staining 24 h post transplantation by confocal microscopy. For comparison with endogenous mitochondria, we stained recipient cells with antibodies against the mitochondrial protein TOMM20. Our results demonstrate that 24 hours after transplantation, transferred mitochondria are localized in similar cellular focal planes as endogenous mitochondria, and that transferred mitochondria are mitotracker red-positive, indicative of an active proton gradient and membrane potential (Fig. 4d). Pre-treatment of cells with H_2_O_2_ enhanced mitochondrial uptake without compromising mitochondrial membrane integrity, further supporting the proficiency to exploit oxidative stress for MitoPlant (Fig. 4d).

### Oxidative stress enhances the survival rate of transplanted mitochondria in retinal ganglion precursor-like cells

**Figure 5:**
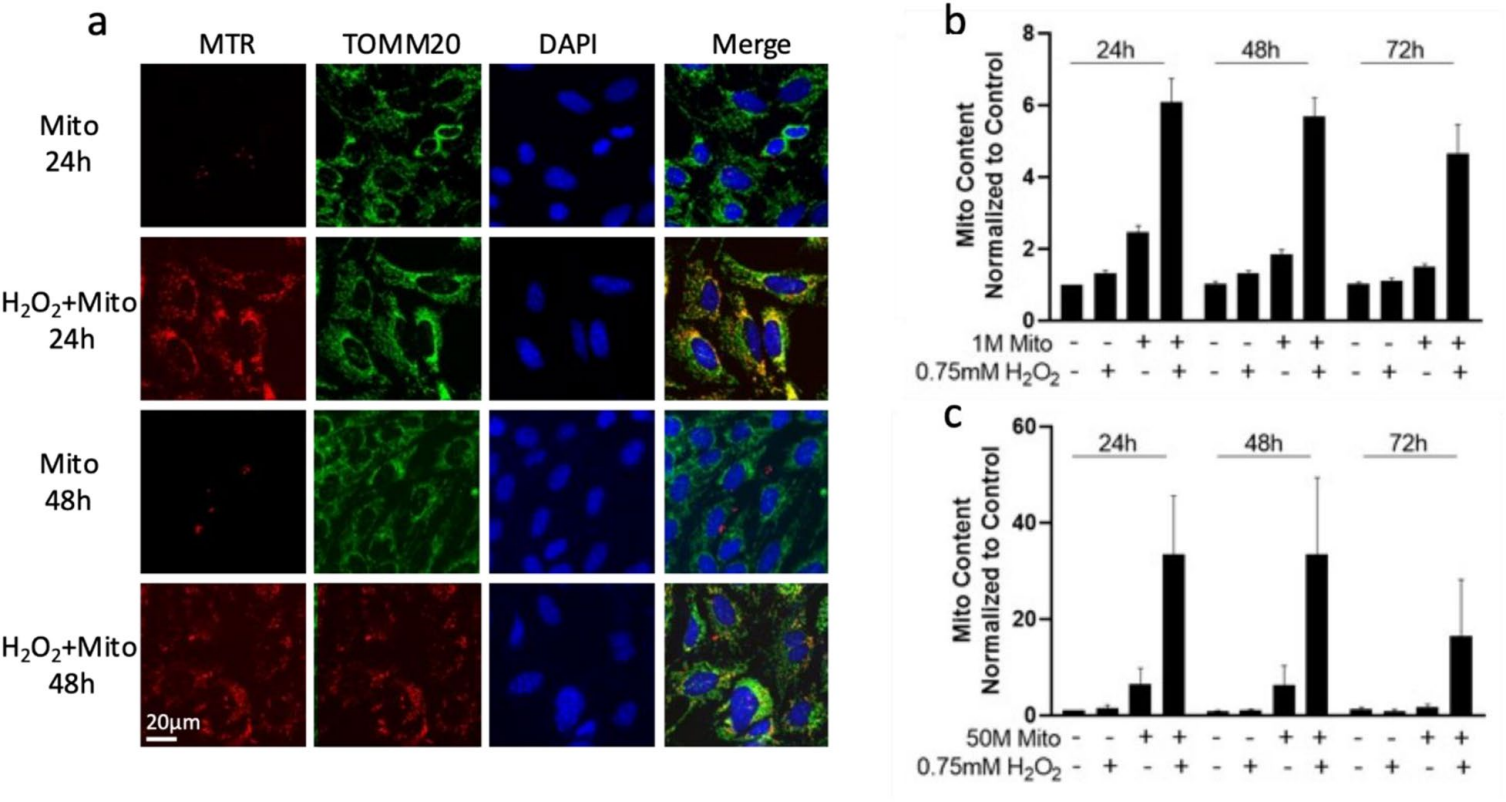
Oxidative stress enhances the survival of the transplanted mitochondria in retinal ganglion precursor-like cells. 661W cells were treated with 0.75 mM H_2_O_2_ for 1 hour and then transplanted with mitochondria isolated from mouse liver. (**a**) Immunofluorescence staining shows exogenous mitochondria (MTR, red) uptake by 661W cells, stained for TOMM20 (endogenous mitochondria marker, green) and for the nuclear marker DAPI (blue) at the indicated time points. (**b**,**c**) Quantitative bar graph of flow cytometry (**b**) and qPCR (**c**) analysis of transplanted mitochondrial survival. Both graphs show a significant increase in mitochondrial content in 661W cells following stress compared to transplanted cells without stress throughout the 72 hours. n = 5–7.

Finally, to assess whether the significant enhancement of mitochondrial uptake by oxidative stress affects their survival in cells, we followed exogenous mitochondria levels for up to 72 hours post transplantation. As demonstrated in Fig. 5a, the exogenous mitochondria (red) can be detected in 661W cells 24 hours and 48 hours following transplantation. Oxidative stress not only increases the transplanted mitochondrial content within the cells, but also expand their distribution in 661W cells, as most of the cells contain exogenous mitochondria. By evaluating staining of mitochondria with MTR following 24 and 48 hours post transplantation, we demonstrated that transplanted mitochondria remain functional with an intact membrane potential over time, both under steady state and when transplantation followed pre-exposure of cells to oxidative stress (Fig. 5a, Supplementary Fig. 4). We also examined the exogenous mitochondrial content using flow cytometry (Fig. 5b) and qPCR (Fig. 5c). Our results demonstrate that after exposure to H_2_O_2_, transplanted mitochondria are detected in recipient cells even after 72 hours, indicating that oxidative stress significantly enhances exogenous mitochondrial survival in the cells.

## Discussion

MitoPlant has been shown to protect damaged tissue in vivo from acute-ischemic insults by increasing oxygen consumption, ATP production, and calcium buffering^9–11^. However, the mechanisms by which MitoPlant confers protection and the dynamics of mitochondrial uptake and survival in recipient cells is not yet understood. In this study, we examined MitoPlant dynamics under normal and oxidative-stress conditions in retinal ganglion precursor-like cells.

Our results show that in vitro, MitoPlant leads to dose-dependent internalization of exogenous mitochondria into the retinal ganglion precursor-like cell-line 661W (Figs. 2, 3, 4, 5). We demonstrate that mitochondria maintain an intact membrane potential in recipient cells over time following transplantation. Interestingly, fluorescence microscopy images show that the transplanted mitochondria are not distributed evenly within target cells, as some cells are positive for exogenous mitochondria and others are negative (Fig. 2, 5), suggesting that spontaneous mitochondrial internalization may be selective.

Notably, exposure of 661W cells to moderate oxidative stress prior to MitoPlant significantly increased mitochondrial uptake by the cells (Fig. 4). This is of importance as oxidative stress has been shown to contribute not only to acute ocular ischemic insults, but also to the progression of other common chronic sight-threatening conditions such as glaucoma^29,30^, AMD^31^ and diabetic retinopathy^32^. The retina is chronically exposed to oxidative stress due to the constant exposure to light and the high oxygen consumption rate of retinal cells and in particular photoreceptor and retinal ganglion cells. Therefore, exploring the dynamics of MitoPlant in retinal cells under conditions of oxidative stress is key.

It has been documented that stress facilitates endogenous mitochondrial transfer between cells, such as transfer of healthy mitochondria to damaged cells^2–4^, or transfer of dysfunctional mitochondria from RGCs to astrocytes in the retina for mitophagy^33^. Yet, to the best of our knowledge, this is the first evidence demonstrating that oxidative stress enhances exogenous mitochondrial uptake into recipient cells. We show that H_2_O_2_ significantly increases exogenous mitochondrial content in cells both by raising the percent of cells that uptake mitochondria, as well as by increasing the levels of exogenous mitochondria in the cells (Figs. 4, 5). The effect of oxidative stress on mitochondrial uptake was independent of membrane permeabilization and cell death (Supplementary Fig. 3), and suggests that oxidative stress may be exploited in the process of MitoPlant to facilitate uptake without compromising cell viability. We further found that the effect of oxidative stress on mitochondrial uptake is not additive, as increasing H_2_O_2_ concentration from 0.5 to 1 mM did not increase mitochondrial uptake.

These findings imply that oxidative stress induces a mechanism that allows for increased mitochondria internalization, yet suggests that the amount of mitochondria that can be internalized by cells under these conditions is limited. Nevertheless, it is plausible to assume that additional mechanisms may further regulate mitochondria uptake dynamics and work either independently or in concert with oxidative stress. Altogether, this set of experiments suggests that there is an intracellular ‘switch’ that controls whether the exogenous mitochondria would be internalized by the cells or not, and also regulates the number of mitochondria that can enter the cell. Indeed, it has been suggested that extracellular transplanted mitochondria promote a signaling cascade within the damaged cells before entering the cells^34,35^, indicating that there may be additional factors that contribute to the protective effect of MitoPlant that are beyond the internalization of an active exogenous mitochondria into the damaged cells. Further studies that will compare the effect of naïve and stressed conditions on MitoPlant dynamics may shed light on the signaling pathways activated by exogenous mitochondria and the mechanisms by which MitoPlant confers a cell-protective effect.

It is still not clear what is the fate of the transplanted mitochondria and how long mitochondria survive in recipient cells. It has been shown that exogenous mitochondria that had been rapidly internalized by cardiomyocytes  (two hours after transplantation) undergo mitophagy and degradation within a few hours, while only some fuse with endogenous mitochondria. It will be interesting to further explore the uptake dynamics and fate of transplanted mitochondria at later time points of transplantation and allude to the mechanisms that dictate the cellular response that is initiated by exposure to exogenous mitochondria. As one of the challenges is to distinguish between the endogenous and exogenous mitochondria, other groups have been using in addition to fluorescent probes xenogeneic MitoPlant^37^ or iron-labeled mitochondria^38^, while others transplanted mitochondria to ρ^0^ cells that lack their own mtDNA^13–15^. However, fluorescent probes may degrade after a few days, iron-labeled mitochondria may lose the iron, and different mitochondrial origins, as well as lack of endogenous mtDNA, may affect the survival of transplanted mitochondria.

Here, we developed a new approach to follow exogenous mitochondria survival based on a difference of two nucleotides in the mtDNA sequence of C57BL/6 and BALB/c mouse strains. Using this technique, we were able to demonstrate a dose dependent increase in mitochondrial content already 3 hours after MitoPlant (Fig. 3c), findings which are in accordance with studies by the group of McCully^36^. In addition, we show that 24 hours after transplantation, there is a significant decrease in the exogenous mitochondrial content (Fig. 3c). This was confirmed also by flow cytometry and microscopy (Fig. 5), indicating that the transplanted mitochondria survive for ~ 24 hours in the recipient cells.

Interestingly, exposure of the recipient cells to moderate levels of H_2_O_2_ before MitoPlant enhanced the survival of the transplanted mitochondria. In Fig. 5 we show that when transplanted following exposure to oxidative stress, exogenous mitochondria can be detected even 72 hours after transplantation, whereas no mitochondria could be detected in this time in transplanted naïve cells (Fig. 5).

Altogether, by developing a new tool to follow exogenous mitochondria in recipient cells, we show that the exogenous mitochondria survive for only ~ 24 hours following transplantation. Further, we found that moderate oxidative stress enhances exogenous mitochondria uptake and survival in retinal ganglion precursor-like cell-line. Understanding the factors that affect the fate of transplanted mitochondria will promote the optimization of MitoPlant as a therapeutic modality, and open the doors to the development of novel treatments for acute insults as well as chronic and genetic mitochondrial-related diseases.

## Supplementary Information

Supplementary Information.

## Data Availability

The datasets generated during and/or analyzed during the current study are available from the corresponding author on reasonable request.
