# Supplementary material for: Oxidative stress facilitates exogenous mitochondria internalization and survival in retinal ganglion precursor-like cells
Source: Sci Rep. 2022 Mar 24;12:5122. doi: 10.1038/s41598-022-08747-3 (PMC8948238; doi:10.1038/s41598-022-08747-3)
Supplement: Supplementary file 1 — Supplementary Information. [file 41598_2022_8747_MOESM1_ESM.pdf]

Oxidative stress facilitates exogenous mitochondria internalization and survival in retinal ganglion precursor-like cells

Supplementary information

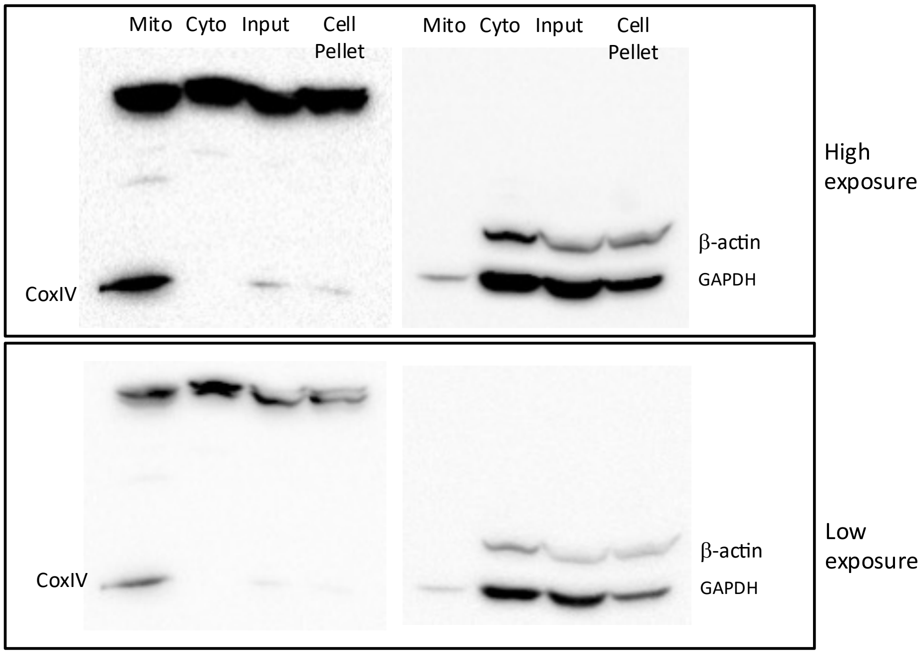

Supplementary Figure 1: Purification of mitochondrial fraction. Mitochondria were isolated from mouse liver. Left: Western blot analysis showing clean mitochondrial fraction (mito), enriched with the mitochondrial marker CoxIV compared to input (cell homogenate) and cell pellet fractions, as expected. Right: the same blot was probed with antibodies to the cytosolic markers β-actin and GADPH, demonstrating that both markers appear in the cytosolic (cyto), input and cell pellets fractions, but not (β-actin) or significantly lower (GAPDH) in the mitochondrial fraction.

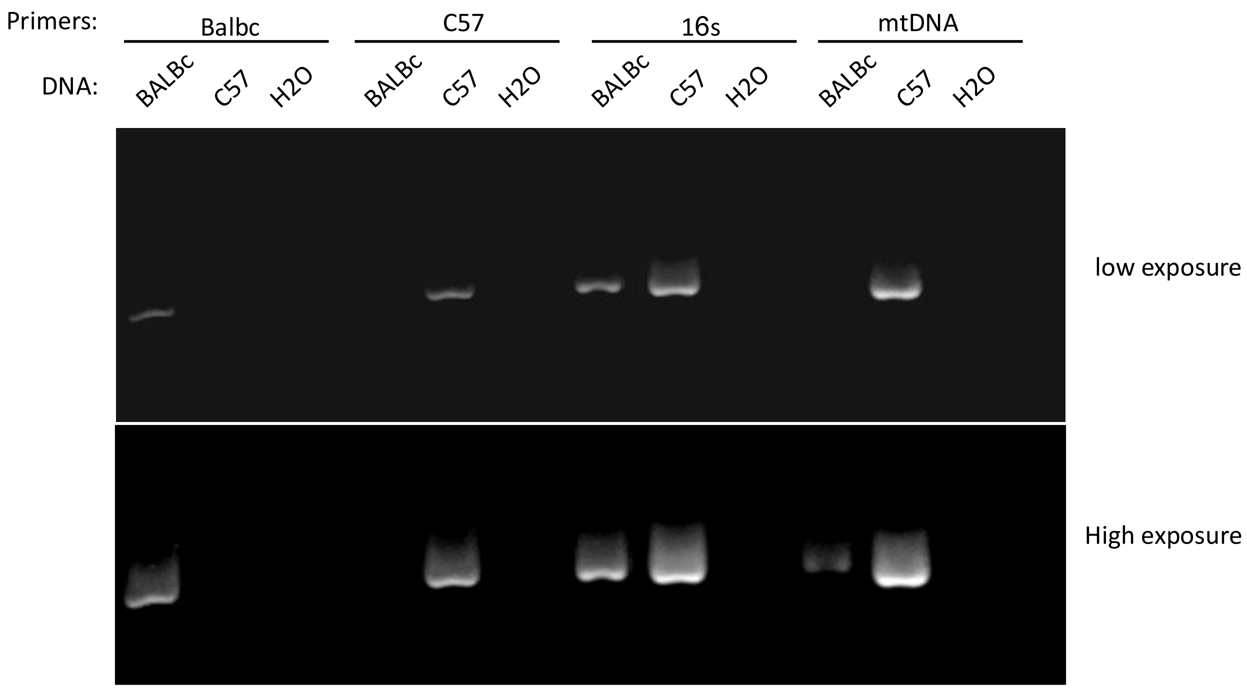

Supplementary Figure 2: Specific amplification of mtDNA from C57BL/6 or BALB/c mice. Mitochondria were isolated from C57BL/6 or BALB/c mice and DNA was extracted. PCR was performed using DNA from each mouse, as indicated above the bands, or DDW (H2O) as control, using primers specific for either BALB/c or C57BL/6. Primers for 16S and mtDNA were used as controls.

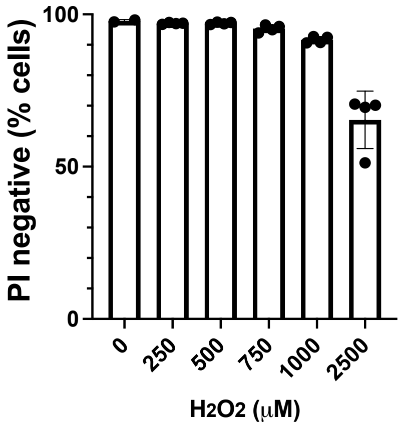

Supplementary Figure 3: Oxidative stress at the conditions used for mitochondrial transplantation does not induce membrane permeabilization. 661W cells were treated with the indicated concentrations of H_2O_2 for one hour and evaluated by flow cytometry for propidium iodide exclusion.

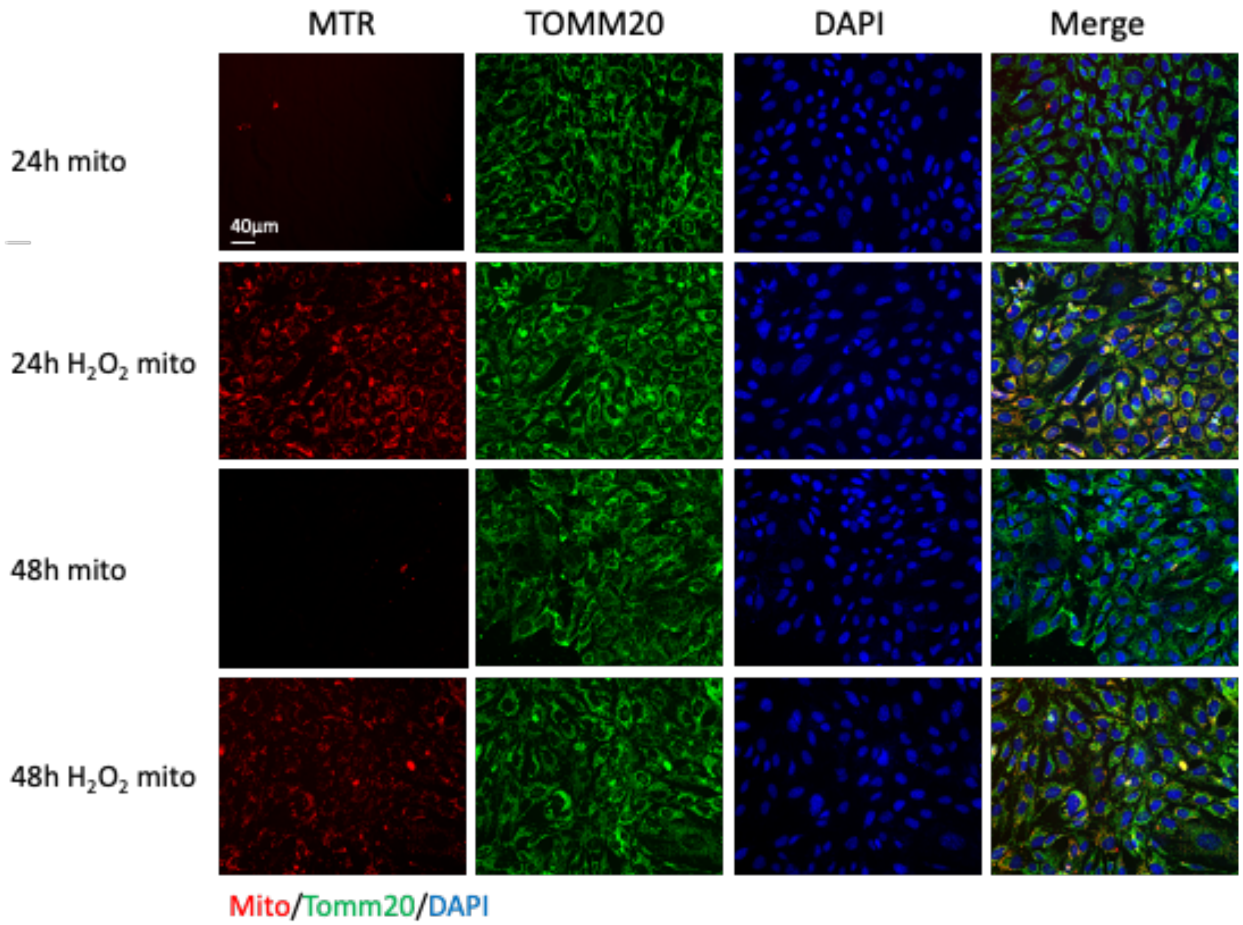

Supplementary Figure 4. Transplanted mitochondria in recipient cells have an intact membrane potential. Mitochondria were isolated from mouse liver and stained with (Mitotracker-red, MTR). Stained mitochondria were then transplanted into 661W cells which were pre-treated with 0.75 mM H_2O_2 for 1h and washed. Endogenous mitochondria in 661W cells were stained with antibodies against TOMM20. Images were taken at 24 and 48 hours post transplantation. DAPI (blue): nuclei.
